# Molecular Abnormalities in Ovarian Cancer Subtypes Other than High-Grade Serous Carcinoma

**DOI:** 10.1155/2010/740968

**Published:** 2009-12-30

**Authors:** C. Blake Gilks

**Affiliations:** Department of Pathology, Vancouver General Hospital, University of British Columbia, Vancouver 1st Floor JPPN, 855 West 12th Ave, Vanouver, BC, Canada V5Z 1M9

## Abstract

Ovarian cancer is the fifth leading cause of cancer death in women in North America, and approximately two-thirds of cases of ovarian cancer are of high-grade serous type. The remaining cases are comprised of a mix of different tumor types (e.g., endometrioid, clear cell, mucinous, etc.), with no single tumor type accounting for more than 10% of ovarian cancer cases. These tumor types can be reproducibly diagnosed, and each features distinct underlying molecular events during oncogenesis, with a characteristic natural history and response rate to conventional cytotoxic chemotherapy. In this review the molecular abnormalities present in the more common non-high-grade serous subtypes of ovarian cancer will be presented. Development of targeted therapies for these tumor types will require understanding of the genetic basis of each tumor type, and may lead to subtype-specific therapy.

## 1. Introduction

Ovarian cancer is not a single disease but is comprised of more than 15 distinct tumor types, each characterized by subtype-specific risk factors (environmental and genetic), precursor lesions, histopathological features, molecular events during oncogenesis, response to chemotherapy, and patient outcome [1, 2] (N.B. the terms “tumor type” and “subtype” are used interchangeably in this paper to refer to the morphologically defined variants of ovarian cancer, as diagnosed in routine surgical pathology practice). More than 90% of ovarian malignancies are carcinomas, commonly referred to as surface epithelial carcinomas, even though there are now significant doubts about the cell of origin of these tumors, and an increasing belief that many, if not most, do not arise from ovarian surface epithelium. Of the group of surface epithelial carcinomas (referred to hereafter simply as carcinomas), approximately 70% are of high-grade serous type [3]. 

High-grade serous carcinomas are chromosomally unstable tumors, and usually have mutations in the TP53 tumor suppressor gene [4]. In most cases they also have germline or somatic mutations in BRCA1 or BRCA2, or promoter methylation of BRCA1 with loss of expression [5]. This underlying loss of BRCA function and inability to repair double-strand repair breaks, leading to chromosomal instability, are an attractive therapeutic target for drugs that target DNA repair (e.g., PARP inhibitors) [6–8].

There is an unfortunate tendency to use the terms “ovarian cancer” and “high-grade serous carcinoma” interchangeably. While this is understandable, given high-grade serous carcinomas account for most cases of ovarian cancer, at least in North America and Europe, and most of the deaths due to ovarian cancer, this has resulted in failure to significantly advance treatment for other ovarian cancer subtypes, particularly the carcinoma subtypes. Although it is current practice to treat all subtypes of carcinoma with the same platinum/taxane chemotherapy, some subtypes do not respond well to this approach and subtype-specific trials of chemotherapy have been recommended for clear cell and mucinous carcinoma in particular [9]. The large randomized clinical trials leading to refinement of the current chemotherapy for ovarian carcinoma have been based on case series that, by current diagnostic criteria, would be composed almost exclusively of high-grade serous carcinomas, and none of these trials permit any conclusions to be drawn about appropriate treatment of other ovarian cancer subtypes. This minority of non-high-grade serous ovarian cancers, consisting of a patchwork of carcinoma subtypes and malignant tumors other than those of surface epithelial type, will be a challenge to study as there are relatively few cases of any given subtype, and large mixed-case series are not appropriate to explore targeted or subtype-specific therapies for these subtypes.

Targeted therapy for ovarian carcinoma, if defined as a therapy directed specifically at molecular abnormalities in individual tumors, will probably require consideration of the tumor subtype, as the molecular abnormalities underlying each of these subtypes are different. The aim of this paper is to present the most common subtypes of ovarian cancer apart from high-grade serous type, discussing first the clinical significance and then presenting an overview of the molecular abnormalities for each subtype. This paper does not cover histopathology, but an important point is that, with recent advances in diagnostic criteria and development of sensitive and specific immunomarkers, all can be reproducibly diagnosed [2, 10]. This reproducibility is recent and historical case series, or more recent retrospective case series without contemporary slide review, are not useful in understanding these uncommon tumor subtypes as a significant number of cases will have been misclassified [10]. The frequency estimates for each subtype are from our center (British Columbia Cancer Agency, which serves a population of 4.1 million) [3], unless otherwise indicated.

## 2. Endometrioid Carcinoma

Endometrioid carcinomas account for approximately 10% of ovarian carcinomas, with most diagnosed at stage I or II. Historical data on endometrioid carcinomas is not reliable as many tumors that were diagnosed as endometrioid in the past are now known to be high-grade serous carcinomas, based on their immunoprofile [11]. Most endometrioid carcinomas are grade 1 or 2 and there is a strong association with endometriosis. Although one of the most common non-serous subtypes, because they are predominantly low stage and low grade at presentation, the burden of morbidity and mortality associated with this subtype is relatively low. While there is a need for adjuvant chemotherapy for advanced-stage endometrioid carcinomas, there is no data currently available specifically on this subtype, and such data will be hard to aquire given that advanced-stage or recurrent tumors are rare. 

**Figure 1:**
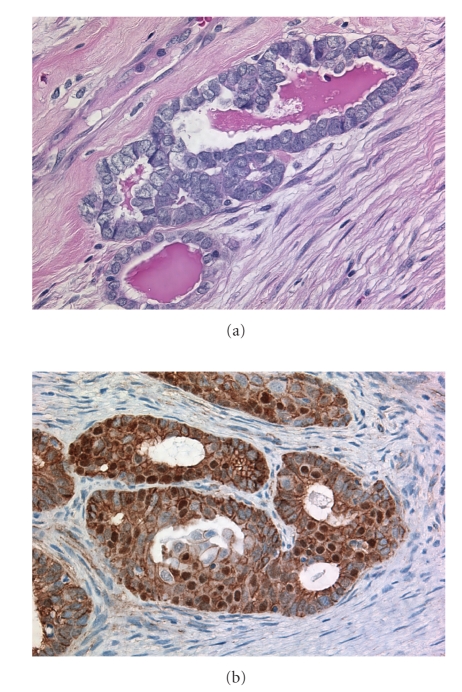
Ovarian carcinoma of endometrioid type (a). Immunostaining for beta-catenin shows both nuclear and membranous localization within the tumor cells (b).

The most common genetic abnormalities in endometrioid carcinoma are somatic mutations in the beta-catenin (CTNNB1) and PTEN genes [12–14]. CTNNB1 mutations are present in 38% to 50% of cases; mutations in codons 32, 33, 37, and 41, of exon 3, involve the phosphorylation sequence for glycogen synthase kinase 3-beta and are thought to lead to decreased APC-mediated downregulation, with accumulation of beta-catenin protein in the nucleus. Nuclear accumulation of beta-catenin protein can be demonstrated in 80% of cases (Figure 1); this contrasts with the exclusively membranous localization seen in other carcinoma subtypes. PTEN is mutated in approximately 20% of cases. BRCA abnormalities and loss of function are not seen in endometrioid carcinomas. Endometrioid carcinomas of the ovary are associated with hereditary nonpolyposis colon cancer syndrome, in patients with germline mutations in a gene encoding a DNA mismatch repair enzyme. This results in microsatellite instability in the tumor cells, which can also occur in sporadic cases as a result of MLH1 promoter methylation. There is coexistence of endometrioid carcinoma of ovary and endometrium relatively frequently (up to 20% of cases of endometrioid carcinoma of the ovary are associated with synchronous atypical hyperplasia or endometrioid adenocarcinoma of the endometrium) [15, 16]. The favorable outcome of such cases suggests that these are independent primaries and also suggests a role of hormonal environment in the genesis of endometrioid carcinoma of the ovary, given the well-characterized role of unopposed estrogenic stimulation as a risk factor for endometrial adenocarcinoma of endometrioid type. Virtually all endometrioid carcinomas of the ovary express estrogen receptor protein [10]. 

## 3. Clear Cell Carcinoma

Clear cell carcinomas occur at a similar frequency as endometrioid carcinomas, and account for approximately 10% of ovarian carcinomas in North America. They are more common in Japan, at least relatively, although this may reflect only a proportional increase, with fewer high-grade serous carcinomas. Clear cell carcinomas also usually present with low-stage disease. All clear cell carcinomas are considered high-grade [1], and they would all be treated with adjuvant chemotherapy in most centers, because of a significant likelihood of relapse, but the available evidence suggests that responses to adjuvant platinum/taxane chemotherapy are uncommon [17–22]. The range of reported response rates is wide (15%–45%), and it is likely that this reflects differences in diagnostic accuracy historically, rather than biological differences in cases series, although there is no proof of this. Because of this poor response rate, and the relatively aggressive nature of clear cell carcinoma, there is an acute need for more effective treatments. Clear cell carcinomas were a subtype specifically mentioned at a recent National Cancer Institute State of the Science meeting on ovarian cancer as being a priority for subtype-specific trials of novel therapeutic agents, in an attempt to identify more effective treatment [9].

The molecular origins of clear cell carcinomas remain obscure. They are not associated with germline or somatic BRCA mutations and typically do not show the complex karyotypes associated with chromosomal instability [5]; most clear cell carcinomas are diploid or tetraploid (B. Risberg and C. B. Gilks, unpublished data). Clear cell carcinomas show relatively low-mitotic rates [5, 23], and it is therefore not surprising that responses to agents targeting dividing cells are less successful than those in high-grade serous carcinoma. Clear cell carcinomas, like endometrioid carcinomas, are strongly associated with the presence of endometriosis and are not uncommonly seen arising in endometriotic cysts. Unlike endometrioid carcinomas, however, they lack expression of hormone receptors (estrogen receptor or progesterone receptor) [24], suggesting that the hormonal influence during oncogenesis is different; clear cell carcinomas may be analgous to the nonhormonally dependent Type 2 endometrial carcinomas while endometrioid carcinomas share many features (both morphological and molecular) with Type 1 carcinomas of the endometrium [25]. Clear cell carcinomas of the ovary show striking similarities to renal clear cell carcinomas, based on gene expression profiling [26], raising the possibility that responses to treatment could be similar in clear cell carcinomas arising at different sites. To investigate this possibility, we treated mice carrying xenografts of an ovarian clear cell carcinoma with sunitinib, a kinase inhibitor that targets VEGF action that is approved for use in patients with renal clear cell carcinoma, and demonstrated a response to sunitinib in the clear cell carcinoma xenograft but not in xenografts derived from three different high-grade serous carcinomas (Y. Z. Wang and C. B. Gilks, unpublished data). Clear cell carcinomas have not been specifically studied for sensitivity to agents targetting angiogenesis/VEGF in human patients, but this may prove to be a fruitful avenue of study. Interestingly, there is evidence that ovarian clear cell carcinomas are sensitive to radiotherapy [27]; given that clear cell carcinomas are not rapidly proliferating tumors, this may reflect targetting of intratumoral neovascularization by the radiotherapy.

## 4. Mucinous Carcinoma

Mucinous carcinomas are much less common than was previously thought, as historically many case series included cases of metastic carcinoma with mucinous differentiation, that were primary in gastrointestinal or biliary tract. Only 3%-4% of ovarian carcinoma are of mucinous type and most are confined to the ovary at presentation. Nonetheless, some will recur and when they do, there are no effective treatments. Mucinous carcinomas, like clear cell carcinomas, were singled out as being a priority for subtype-specific clinical trials, given the ineffectiveness of current therapy [9].

**Figure 2:**
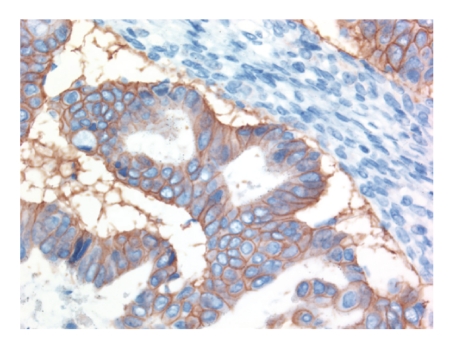
HER2 immunostaining of a mucinous carcinoma shows diffuse membranous positivity. This was associated with high-level HER2 amplification on FISH analysis.

Mutations in KRAS, involving codons 12 and 13, are the most common mutations described in mucinous carcinomas [28]. Mutations can be seen in benign-appearing areas of mucinous tumors, adjacent to frank mucinous carcinoma, suggesting that they are an early event during oncogenesis. HER2 amplification, with overexpression of the protein on the membrane of the tumor cells, is present in 15%–20% of mucinous carcinomas of the ovary (J. N. McAlpine et al. BMC Cancer, in press.) (Figure 2). This is a higher frequency of HER2 amplification than is seen in breast cancer, and it is similar to the frequency encountered in adenocarcinoma of the gastroesophageal junction. Trastuzumab (Herceptin) therapy is an obvious treatment choice for these cases but there is no data yet on response of mucinous carcinomas of the ovary with HER2 amplification/overexpression to such treatment. Although there are large clinical trials of trastuzumab therapy in ovarian cancer, with discouraging results, almost all tumors in these studies were of high-grade serous type [29]. High-grade serous carcinomas only rarely show high-level amplification of the HER2 gene or overexpression of HER2 protein on the cytoplasmic membrane of tumor cells [30], and these studies are not informative about efficacy of this therapeutic option in mucinous carcinoma. 

## 5. Low-Grade Serous Carcinoma

The separation of serous carcinomas into low-grade and high-grade types is a recent development. Comparison of low-grade and high-grade serous carcinomas shows that the low-grade serous carcinomas often arise from a serous borderline tumor while the precursor lesion of high-grade serous carcinoma is tubal intraepithelial carcinoma, in most cases. Low-grade serous carcinomas can be reproducibly distinguished from high-grade serous carcinomas, based primarily on their very uniform nuclei, using low-mitotic rate as a secondary diagnostic criterion [31, 32]. Low-grade serous carcinomas are much less common than high-grade serous carcinomas and account for only 2% of ovarian carcinomas. As many present with high-stage disease, however, there is a need for effective chemotherapy. An unusual feature of the natural history of low-grade serous carcinomas is that they may follow a relatively indolent course; this in turn allows for multiple opportunities to treat [33]. The response rate to platinum/taxane chemotherapy within this group is difficult to gauge, as there are no studies of large series of well-characterized cases. In the case of serous borderline tumors that have progressed to low-grade serous carcinomas, however, response rates are relatively low, with most patients showing no response [34]. As is the case for clear cell carcinomas, low-grade serous carcinomas have a low-mitotic rate, and poor response to platinum-based chemotherapy is not unexpected. 

KRAS or BRAF mutations, which target the same molecular pathway, are present in most low-grade serous carcinomas [35–37]. These tumors are also almost invariably positive for hormone receptor expression (estrogen and/or progesterone receptors). Low-grade serous carcinomas are not chromosomally unstable; they are usually diploid or near diploid and do not show the complex genetic abnormalities seen in high-grade serous carcinomas [38]. Low-grade serous carcinomas are not associated with either germline or somatic abnormalities in BRCA1/2 and typically do not have TP53 mutations. Only rarely do low-grade serous carcinomas progress to higher-grade tumors [39].

## 6. Granulosa Cell Tumor

Granulosa cell tumors are the most common malignant tumors within that group of tumors arising from ovarian sex cord or stromal cells, and account for a large majority of the malignant tumors within this category [1]. They are still relatively uncommon, and have been reported to account for approximately 1%-2% of ovarian tumors (benign or malignant), although in our experience this is an overestimate. They may account for 2% of ovarian cancers, however. They are the most common primary ovarian malignancies, apart from carcinomas. As with some of the other subtypes discussed above, they have not been reliably diagnosed in the past, so that older data regarding their natural history or molecular abnormalities is not reliable. There are two distinct granulosa cell variants and the discussion that follows relates only to the adult-type granulosa cell tumors, which account for 95% of granulosa cell tumors. These tumors are usually confined to the ovary at presentation, and recurrences can be many years after presentation [1]. The only effective therapeutic option at present is surgery.

**Figure 3:**
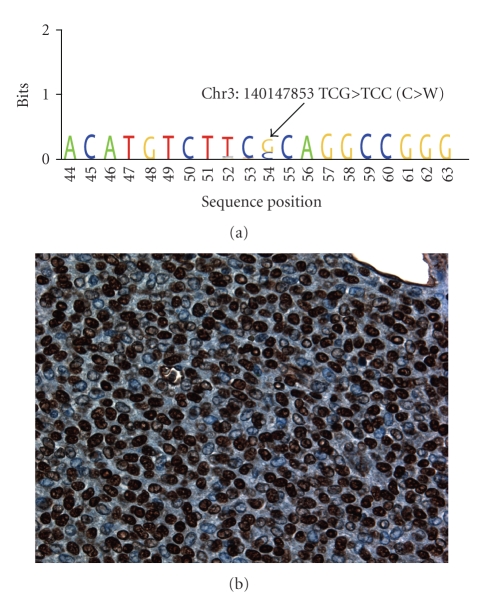
Results of sequencing of the transcriptome of a granulosa cell tumor, showing sequence from the FOXL2 gene (a). At nucleotide 402, both G and C were identified, indicating that this tumor was hemizygous for the 402C->G mutation charateristic of adult-type granulosa tumor. Granulosa tumor cell nuclei show high-level expression of the FOXL2 protein by immunostaining (b), in association with this mutation.

Granulosa cell tumors are genomically stable and diploid. They show few abnormalities by cytogenetic analysis. It is likely that some tumors considered to be aneuploid granulosa cell tumors in the past were undifferentiated carcinomas, based on their natural history (early recurrence and poor prognosis). Recently 4 granulosa cell tumors were subjected to transcriptome sequencing, revealing a missense G > C mutation at nucleotide 402 of the FOXL2 gene in every case (Figure 3) [40]. Extension of this study by examination of additional cases revealed that these identical 402G->C FOXL2 mutations were present in more than 95% of cases diagnosed as adult-type granulosa cell tumors, as well as occasional thecomas, and a single juvenile granulosa cell tumor (of ten tested) [40]. It is likely that at least two of the three cases of purported adult-type granulosa cell tumor lacking an FOXL2 mutation were misdiagnosed and were not granulosa cell tumors, based on a review of the tumor's immunophenotypes, while the thecomas showing the FOXL2 mutation did have minor granulosa cell components, on retrospective review of the cases. 

The FOXL2 gene is a member of the forkhead/winged-helix family of transcription factors, and this point mutation results in a cysteine to tryptophan change at position 134 in the amino acid sequence of the protein, a highly nonconservative change, which is predicted to affect protein-protein interactions. FOXL2 is a crucially important transcription factor in granulosa cell development; an autosomal recessive disorder, blepharophimosis-ptosis-epicanthus inversus syndrome, occurring as a result of two mutant alleles of FOXL2, is associated with ovarian failure [41–43]. In granulosa cell tumors, the FOXL2 mutations are somatic; in all cases tested, the germline sequence has been normal. The near universal presence of this FOXL2 mutation in adult-type granulosa cell tumors, the fact that most tumors are hemizygous for the mutation, and the presence of abundant FOXL2 protein in tumor cell nuclei (Figure 3) suggest that this mutation is a critical genetic abnormality in the genesis of adult-type granulosa cell tumors and that it is an activating mutation. FOXL2 interacts with SMAD and AP1 proteins, and it is possible that this interaction is disrupted, leading to uncontroled growth. The presence of a single mutation suggests the possibility of targeted therapy, similar to what has been developed for other cancers where specific recurrent genetic abnormalities are present (e.g., chronic myelogenous leukemia, gastrointestinal stromal tumor, and dermatofibrosarcoma protuberans).

## 7. Dysgerminoma

Dysgerminomas are within the group of primitive germ cell tumors, which are defined as malignant, nonteratomatous germ cell tumors [1]. Dysgerminomas are morphologically indistinguishable from their much more common counterpart in the male, testicular seminoma. Although dysgerminomas are the most common of the primitive germ cell tumors of the ovary, they are rare and account for less than 1% of ovarian cancers. These tumors are chemosensitive, and most patients, even with advanced-stage disease at presentation, can be cured.

The genetic abnomormalities in dysgerminoma are identical to those of seminoma. Cytogenetically, abnormalities of chromosome 12, particularly i(12p), are commonly present [44]. Activating mutations in KIT are present in a significant minority of patients with dysgerminoma and are associated with high-level expression of KIT protein in the tumor cells [45–47]. KIT protein can also be present in dysgerminomas without an identifiable KIT mutation; the mechanism underlying KIT overexpression in these cases is not known.

## 8. Summary

Although there has been progress in elucidating the molecular basis of the less common subtypes of ovarian cancer, there remains much work to be done if targeted therapy is to become a routine option clinically. There are compounds available that can target some of the molecular abnormalities identified (HER2 amplification in mucinous carcinoma, neovascularization and VEGF signaling in clear cell carcinoma, hormone receptor signaling in low-grade serous carcinoma, and KIT mutations in dysgerminoma); future studies should focus on both identifications of additional targets; rational preclinical studies and subtype-specific clinical trials of targeted therapies aimed at promising molecular targets.
